# Soil Nitrogen Transformation Process Influenced by Litterfall Manipulation in Two Subtropical Forest Types

**DOI:** 10.3389/fpls.2022.923410

**Published:** 2022-07-14

**Authors:** Wende Yan, Taimoor Hassan Farooq, Yi Chen, Wancai Wang, Rubab Shabbir, Uttam Kumar, Muhammad Umair Riaz, Saqer S. Alotaibi, Yuanying Peng, Xiaoyong Chen

**Affiliations:** ^1^National Engineering Laboratory for Applied Technology of Forestry and Ecology in South China, Changsha, China; ^2^Ecological Restoration Innovation Alliance for Southern Purple Shale Mountains, Changsha, China; ^3^College of Life Science, Central South University of Forestry and Technology, Changsha, China; ^4^National Observation Research Station of Forest Ecosystem of Lutou Forest Farm in Hunan Province, Yueyang, China; ^5^Bangor College China, A Joint Unit of Bangor University and Central South University of Forestry and Technology, Changsha, China; ^6^Department of Plant Breeding and Genetics, Seed Science and Technology, University of Agriculture, Faisalabad, Pakistan; ^7^Faculty of Agriculture, Center for Molecular and Functional Ecology in Agroecosystems, University of Talca, Talca, Chile; ^8^Department of Forestry, Range and Wildlife Management, The Islamia University of Bahawalpur, Bahawalpur, Pakistan; ^9^Department of Biotechnology, College of Science, Taif University, Taif, Saudi Arabia; ^10^College of Arts and Sciences, Lewis University, Romeoville, IL, United States; ^11^College of Arts and Sciences, Governors State University, University Park, IL, United States

**Keywords:** litterfall, nitrogen forms, Masson pine forest, camphor tree forest, subtropical region

## Abstract

Nitrogen (N) is often recognized as the primary limiting nutrient element for the growth and production of forests worldwide. Litterfall represents a significant pathway for returning nutrients from aboveground parts of trees to the soils and plays an essential role in N availability in different forest ecosystems. This study explores the N transformation processes under litterfall manipulation treatments in a Masson pine pure forest (MPPF), and Masson pine and Camphor tree mixed forest (MCMF) stands in subtropical southern China. The litterfall manipulation included litterfall addition (LA), litterfall removal (LR), and litterfall control (LC) treatments. The project aimed to examine how litterfall inputs affect the soil N process in different forest types in the study region. Results showed that soil ammonium N (NH_4_^+^-N) and nitrate N (NO_3_^−^-N) content increased under LA treatment and decreased under LR treatment compared to LC treatment. LA treatment significantly increased soil total inorganic N (TIN) content by 41.86 and 22.19% in MPPF and MCMF, respectively. In contrast, LR treatment decreased the TIN content by 10 and 24% in MPPF and MCMF compared to LC treatment. Overall, the soil net ammonification, nitrification, and N mineralization rates were higher in MCMF than in MPPF; however, values in both forests were not significantly different. LA treatment significantly increased annual net ammonification, nitrification, and mineralization in both forest types (*p* < 0.05) compared to LC treatment. LR treatment significantly decreased the values (*p* < 0.05), except for ammonification, where LR treatment did not differ substantially compared to LC treatment. Our results suggested that changes in litterfall inputs would significantly alter soil N dynamics in studied forests of sub-tropical region. Moreover, mixed forest stands have higher nutrient returns due to mixed litter and higher decomposition compared to pure forest stands.

## Introduction

Nitrogen (N) is often recognized as a limiting nutrient element for tree growth in various forest ecosystems (Schimel and Bennett, 2004; Farooq et al., 2021a). Soil N mineralization is defined as the process in which the organic N is converted to inorganic N in the soil through the decomposition processes. It is a vital index determining forest growth, nutrient cycling, and soil fertility in forest ecosystems (LeBauer and Treseder, 2008). N mineralization is crucial in maintaining soil health, regulating site fertility, enhancing biomass productivity, and improving forest ecosystem services (Urakawa et al., 2016). As an essential part of N cycling in forest ecosystems (Farooq et al., 2021b), N mineralization is influenced by different biotic and abiotic factors, including forest types (Hobbie, 2015), quantity and quality of the litterfall (Fierer et al., 2005), site conditions (Cookson et al., 2007; Farooq et al., 2019a), and climatic factors.

Forest types could cause a significant change in the rates and patterns of the N mineralization process (Mgelwa et al., 2019). Differences in litter quantity and quality resulting from various tree species in these forests would alter litter decomposition. For example, Pérez et al. (1998) reported that annual N mineralization rates varied from 20 to 23 kg‧ha^−1^ and 31 to 37 kg‧ha^−1^ for Fitzroya and Nothofagus forest, respectively, in the coastal range of southern Chile. Son and Lee (1997) reported that N mineralization and nitrification were significantly different among the tree species, and the annual N mineralization rate was about 44 kg‧ha^−1^ for *P. rigida*, 92 kg‧ha^−1^ for *Larix leptolepis*, and 112 for *Q. serrata*. Litterfall quantity and quality accumulated on the forest floors were attributed to the variation of soil N dynamics because litterfall strongly influenced environmental factors which regulated soil N transformations.

Serval studies have examined the influence of litter amounts on soil N mineralization processes; however, the effect of litterfall changes on N mineralization and nitrification processes is still not fully understood in different forest types, because the increasing atmospheric CO_2_ concentration could stimulate tree productivity and thereby increase litterfall production in different forest ecosystems (Sayer et al., 2007). Therefore, understanding how these environmental factors regulate soil N transformations in forests is essential for sustainable forest ecosystem management.

The present study aimed to investigate the influence of litterfall manipulation treatments on the soil N transformation process (inorganic N, N mineralization, ammonification and nitrification, and net annual production) in two common but important forest types in subtropical China: Masson pine pure forests (MPPF) and Masson pine and Camphor tree mixed forests (MCMP). Masson pines are important for the ecological balance of forest ecosystems. This species prevents soil erosion because the pine tree’s roots hold the soil in place (Gilani et al., 2020). Whereas apart from providing attractive red and yellow striped wood, the Camphor tree has various medicinal properties.

We hypothesized that (1) sites receiving more litterfall would have higher N mineralization and nitrification rates than sites that received less litterfall, and (2) the MCMP stands would have a higher N transformation rate than the MPPF stands due to mixed litterfall.

## Materials and Methods

### Study Sites

The study was performed at Hunan Botanical Garden in Changsha city, China, Hunan Province. It is a typical moist subtropical zone with a mean annual temperature of 17.2°C and mean temperatures of 4.7 and 29.4°C in January and July, respectively. Annual precipitation ranged between 1,200 and 1700 mm with an average annual rainfall of 1,422 mm, most from April to August. The mean annual relative humidity was >80%. The frost-free period was 270–310 days per year. The garden area covers about 140 ha, and the elevation is 50–110 m with an average site slope of 5–15^o^. According to US soil taxonomy, the soil is classified as typical red earth, and the soil texture ranges from clay loam to sandy loam. Soil pH on the surface (0–10 cm) was acidic, with an average pH of 5.0. The dominant tree species in the garden are Camphor tree [*Cinnamomum camphora* (L.) J. Presl.], Chinese fir [*Cunninghamia lanceolata* (Lamb.) Hook.], Chinese sweet gum (*Liquidambar acalycina* H.T. Chang), Masson pine (*Pinus massoniana* Lamb.), and Slash pine (*Pinus elliottii* Engelm.). The major understory plants included *Camptotheca acuminate* Decne*, Clerodendrum cyrtophyllum* Turcz, *Litsea mollis* Hemsl, *Cyclobalanopsis glauca*, *Camellia oleifera*, *Castanopsis sclerophylla* (Lindl. and Paxton) Schottky, *Lophatherum sinense* Rendle, and *Phytolacca acinosa* Roxb.

### Experimental Design

**Figure 1:**
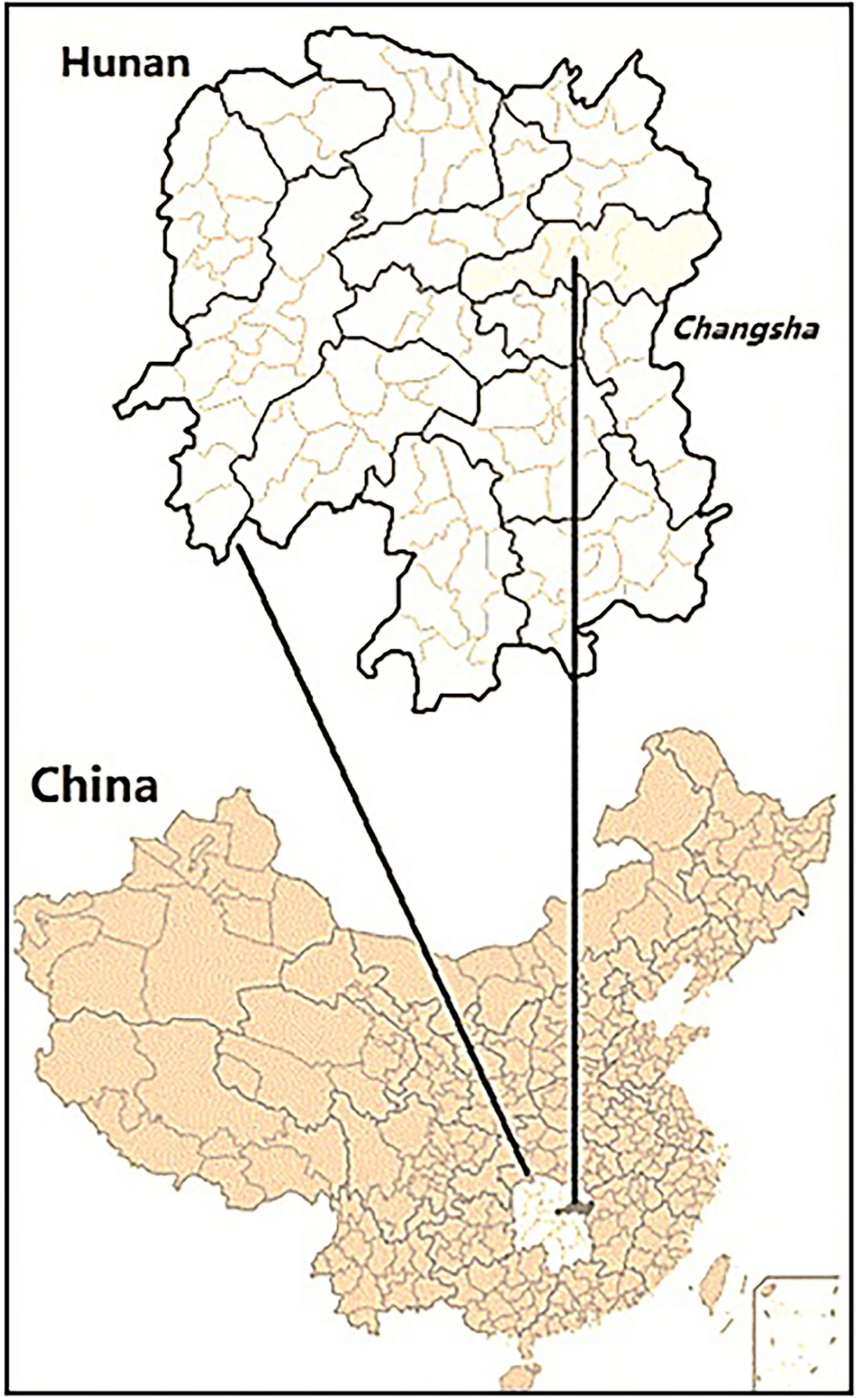
Location of the study area, Hunan Botanical Garden in Changsha city, Hunan Province, China (113°02′-113°03′ E, 28°06′-28°07′ N).

Masson pine pure forests (MPPF) and Masson pine and Camphor tree mixed forests (MCMP) were selected as research plots. Both MPPF and MCMF stands were planted in 1991. The initial tree density was 2 m × 2 m in MPPF stands, and 2 m × 3 m in MCMF stands, with a proportion of 50%:50% of the Masson pine and Camphor tree species in the stands. The experiment was set up as a split-plot design, with the main factor as forest types. Four replication plots with 20 m × 20 m were established in each forest type. Litter treatments as the sub-factors nested in the forest types. The litter traps were designed as 2 × 3 m square in size using a nylon netting screen (mesh size 1 mm). Using ropes, the litter traps were mounted on wooden poles approximately 80 cm above the ground. Three replications of litter traps were randomly set up in each plot for collecting the litterfall. The characteristics of the selected two forest types in the study area are shown in Table 1 (Figure 1).

**Table 1:** Characteristics of the two selected forest types in the study site (DBH = Diameter at breast height).

Forest type	Stand density (trees ha^−1^)	Stand age (year^−1^)	Mean DBH (cm)	Average height (m)	Under crown height (m)	Canopy density
MPPF^*^	1324	25	16.9 ± 2.32	12.7 ± 2.23	7.8 ± 3.23	0.8
MCMF	1356	25	16.2 ± 2.94	12.3 ± 3.12	6.2 ± 3.26	0.9

* MPPF, Masson pine pure forests; MCMF, Masson pine and Camphor tree mixed forests.

### Litterfall Manipulation Treatments

In this study, litterfall manipulation contained three treatments: litterfall removal (LR), litterfall addition (LA), and natural litterfall input (as litter control, LC). The LR treatment was performed by removing all the litter materials from the forest floor in the study plot. Then, the litter traps were installed in the plot to prohibit litter from falling on the floor. All the litter was collected and removed from the mesh traps on a 2-week basis. The LA treatment was performed to transfer and evenly distribute litter materials obtained from an LR plot described above on a LA plot. The LA treatment was carried out at the time when LR was performed. The LC treatment was performed to keep the natural status of litterfall on the floor, and the normal litterfall process was allowed, neither removal nor addition in the plots.

### Soil Sample Collections

Soil samples were collected from all litterfall treatment plots in February, May, August, and November 2016. Approximately 100 g of mineral soils was collected from 0 to 15 cm soil depth from each of the five locations in a plot about 1 m apart. The soil samples from these five points were pooled to form a soil composite sample. The soil samples were loaded into plastic bags and delivered to the Central South University of Forestry and Technology (CSUFT) laboratory for further analysis.

### Soil N Transformation Dynamics Measurements

Soil NH_4_^+^-N and NO_3_^−^-N contents were measured using a Flow Injection Auto Analyzer (Lachat Instruments, Milwaukee, WI; Son et al., 1995). Briefly, 10 g of 2-mm sieved and field-moist soils with 100 ml of 2 M KCl on a glass bottle was shaken for 60 min on a reciprocating shaker. Then, the extracts were settled for 30 min. After that, the extracts were passed through a Whatman #42 filter paper. These extracts were analyzed for NH_4_^+^-N and NO_3_^−^-N concentrations.

Using a resin core method, soil net N mineralization was measured four times in 2016 (February, May, August, and November; Hatch et al., 2000). Undisturbed soil cores were isolated and incubated inside PVC cylinders (4.0 cm in diameter, 12 cm high, equipped with 5 g anion exchange resin). Two PVC cylinders were used for each N mineralization measurement time in each litterfall manipulation plot. One PVC cylinder was used for *in situ* incubation, and the other was used to take soil samples. At the beginning and end of each incubation period, the soil NH_4_^+^-N and NO_3_^−^-N measurements were used to calculate soil N mineralization rate, soil net N ammonification rate, and soil net N nitrification rate under different litterfall treatments in the examined forests.


(1) Soil net N ammonification rate = Soil NH4+-N content after incubation − Soil NH4+-N content before incubation/30 days of incubation

(2) Soil net nitrification rate = Soil NO3−-N content after incubation − Soil NO3−-N content before incubation/30 days of incubation

(3) Soil net N mineralization rate = Soil inorganic N content after incubation − Soil inorganic N content before incubation/30 days of incubation

No N was assumed to be lost for denitrification (Giardina et al., 2001). Annual net N ammonification, net nitrification, and net mineralization amount were calculated by summing these N values of incubation periods spanning a year (Son and Lee, 1997; Yoon et al., 2015).

### Data Analysis

Two-way ANOVA was performed to statistically test the effects of forest types (Masson pine forest and the Mixed forest), litterfall manipulation treatment (LA, LR and LC), and their interactions on characteristics of soil N transformations. Log transformations were done for the original soil net ammonification, nitrification, and mineralization data to satisfy the normality and homoscedasticity assumptions of ANOVA. Pair-wise t-tests were used to examine differences between two types of forest stands, and then the means of LA, LR, and LC were compared by a Tukey–Kramer test within each forest type. Statistical analyses were conducted using the SAS statistical package (SAS Institute, Inc., Cary, NC 1999–2001).

## Results

The average soil NH_4_^+^-N and NO_3_^−^-N and total inorganic N contents in LC plots were significantly higher in MCMF than in MPPF (*p* = 0.036; Table 2). LA treatment increased soil NH_4_^+^-N content by 23% in MPPF and about 18% in MCMF compared to LC; however, the differences in NH_4_^+^-N content between LA and LC treatments were not significantly different (*p* = 0.058). LR treatment significantly declined soil NH_4_^+^-N content in MCMF (*p* = 0.048), but not in MPPF (*p* = 0.061). In contrast, LA treatment significantly increased soil NO_3_^−^-N content by about 50 and 25% in MPPF and MCMF, respectively, compared to LC treatment (*p* = 0.021, Table 2). Soil NO_3_^−^-N content was reduced by 15–20% in LR plots in both forest types, but this reduction was not significant compared to LC plots (*p* > 0.05). In terms of total inorganic N content, overall, it was higher (33.15 ± 5.95 mg‧kg^−1^) under LA treatment in the MCMF forest stand. LA treatment significantly increased soil total inorganic N content (about 41%), while LR treatment decreased (about 10%) compared to LC treatment in MPPF. Same as MPPF, in MCMF, both LA and LR treatments significantly altered total inorganic N content (*p* = 0.047) compared to LC, with the former increasing it by 22% and the latter by 24% (Table 2).

**Table 2:** Changes in the content of soil nitrogen forms under different litterfall manipulation treatments in two sub-tropical forest types.

N form		Treatment	MPPF		MCMF	
		Content (mg‧kg^−1^)	Change (%)	Content (mg‧kg^−1^)	Change (%)
NH_4_^+^-N	LA^*^	9.66 ± 2.94 Ab	23.21↑	14.07 ± 3.93 Aa	18.24↑
	LC	7.84 ± 1.29 Ab	0.00	11.90 ± 2.42 Aa	0.00
	LR	7.79 ± 1.01 Aa	0.64↓	7.94 ± 1.88 Ba	33.28↓
NO_3_^−^-N	LA	15.23 ± 4.81 Aa	49.90↑	19.08 ± 8.75 Aa	25.28↑
	LC	10.16 ± 4.45 Bb	0.00	15.23 ± 6.79 Ba	0.00
	LR	8.17 ± 3.19 Bb	19.59↓	13.00 ± 6.48 Ba	14.64↓
Total inorganic N	LA	24.89 ± 4.42 Ab	40.86↑	33.15 ± 5.95 Aa	22.19↑
	LC	17.67 ± 4.94 Bb	0.00	27.13 ± 4.64 Ba	0.00
	LR	15.97 ± 3.08 Bb	9.62↓	20.73 ± 5.22 Ca	23.59↓

* LA, litter addition; LC, litter control; LR, litter removal; MPPF, Masson pine pure forests; MCMF, Masson pine and Camphor tree mixed forests. Values are mean ± SD. Different capital letters indicate significant differences among the same forest type column treatments. In contrast, lower-case letters indicate significant differences between the forest types under the same treatment (*p* < 0.05). Upper ↑ *indicates a positive change, and down* ↓ indicates a negative change in LA and LR treatments compared to LC treatment.

Overall, the soil net N ammonification rate, nitrification rate, and mineralization rates were higher in MCMF than in MPPF; however, their specific values in both forests were not significantly different (*p* = 0.054; Table 3). LA treatment significantly increased soil ammonification rate, nitrification rate, and mineralization rate by 8.54, 13.66, and 14.29% in MPPF, respectively; on the contrary, LR treatment decreased these rates significantly, except for the soil net ammonification, where the rate did not differ considerably between LR plots and LC plots in MPPF. In MCMF, soil ammonification rate, nitrification rate, and mineralization rate significantly increased by 56.80, 35.08, and 38% in LA plots. These rates were significantly reduced by 34% in LR plots compared to the LC plots (Table 3). No significant differences were found in annual net ammonification, nitrification, and mineralization amount in LC plots in the studied forests (*p* < 0.05; Table 3).

**Table 3:** Variations in the soil nitrogen transformation processes under different litterfall manipulation treatments in two sub-tropical forest types.

N form	Treatment	MPPF		MCMF	
		Rate (mg‧kg^−1^‧d^−1^)	Change (%)	Rate (mg‧kg^−1^‧d^−1^)	Change (%)
Net Ammonification rate	LA^*^	0.30	8.54↑	0.42	56.80↑
	LC	0.26	0.00	0.27	0.00
	LR	0.24	1.98↓	0.18	33.58↓
Net Nitrification rate	LA	0.99	13.66↑	0.34	35.08↑
	LC	0.75	0.00	0.25	0.00
	LR	0.28	29.56↓	0.15	34.06↓
Net N mineralization rate	LA	1.29	14.29↑	0.76	38.00↑
	LC	1.01	0.00	0.52	0.00
	LR	0.52	26.01↓	0.33	33.99↓

* LA, litter addition; LC, litter control; LR, litter removal; MPPF, Masson pine pure forests; MCMF, Masson pine and Camphor tree mixed forests. Upper ↑ *indicates a positive change, and down* ↓ indicates a negative change in LA and LR treatments compared to LC treatment.

Among all the treatments, annual NH_4_^+^-N was higher under LA treatment and lowest under LR treatment in the MCMF forest. For the remaining treatments, the difference was not significantly different for both forest stands (*p* = 0.052). The same patterns go for annual NO_3_^−^-N and annual total inorganic N production; however, significant differences were observed among all the studied treatments (*p* = 0.039; Table 4).

**Table 4:** Annual production of different nitrogen forms under litterfall manipulation treatments in MPPF and MCMF stand.

N form	Treatments	MPPF	MCMF
		Annual N production (mg‧kg^−1^‧yr.^−1^)	Annual N production (mg‧kg^−1^‧yr.^−1^)
Annual NH_4_^+^-N	LA^*^	35.99 ± 1.29 Ab	50.99 ± 1.29 Aa
	LC	30.36 ± 1.29 Ba	32.52 ± 1.29 Ba
	LR	29.76 ± 1.29 Ba	21.60 ± 1.29 Cb
Annual NO_3_^−^-N	LA	233.89 ± 1.29 Ab	283.68 ± 1.29 Aa
	LC	205.78 ± 1.29 Ba	210.01 ± 1.29 Ba
	LR	144.96 ± 1.29 Ca	138.48 ± 1.29 Ca
Annual total inorganic N	LA	269.88 ± 1.29 Ab	334.68 ± 1.29 Aa
	LC	236.14 ± 1.29 Ba	242.52 ± 1.29 Ba
	LR	174.72 ± 1.29 Ca	160.08 ± 1.29 Ca

* LA, litter addition; LC, litter control; LR, litter removal; MPPF, Masson pine pure forests; MCMF, Masson pine and Camphor tree mixed forests. Values are mean ± SD. Different capital letters indicate significant differences among the treatments in the same forest type column. In contrast, lower-case letters indicate significant differences between the forest types under the same treatment (*p* < 0.05).

**Figure 2:**
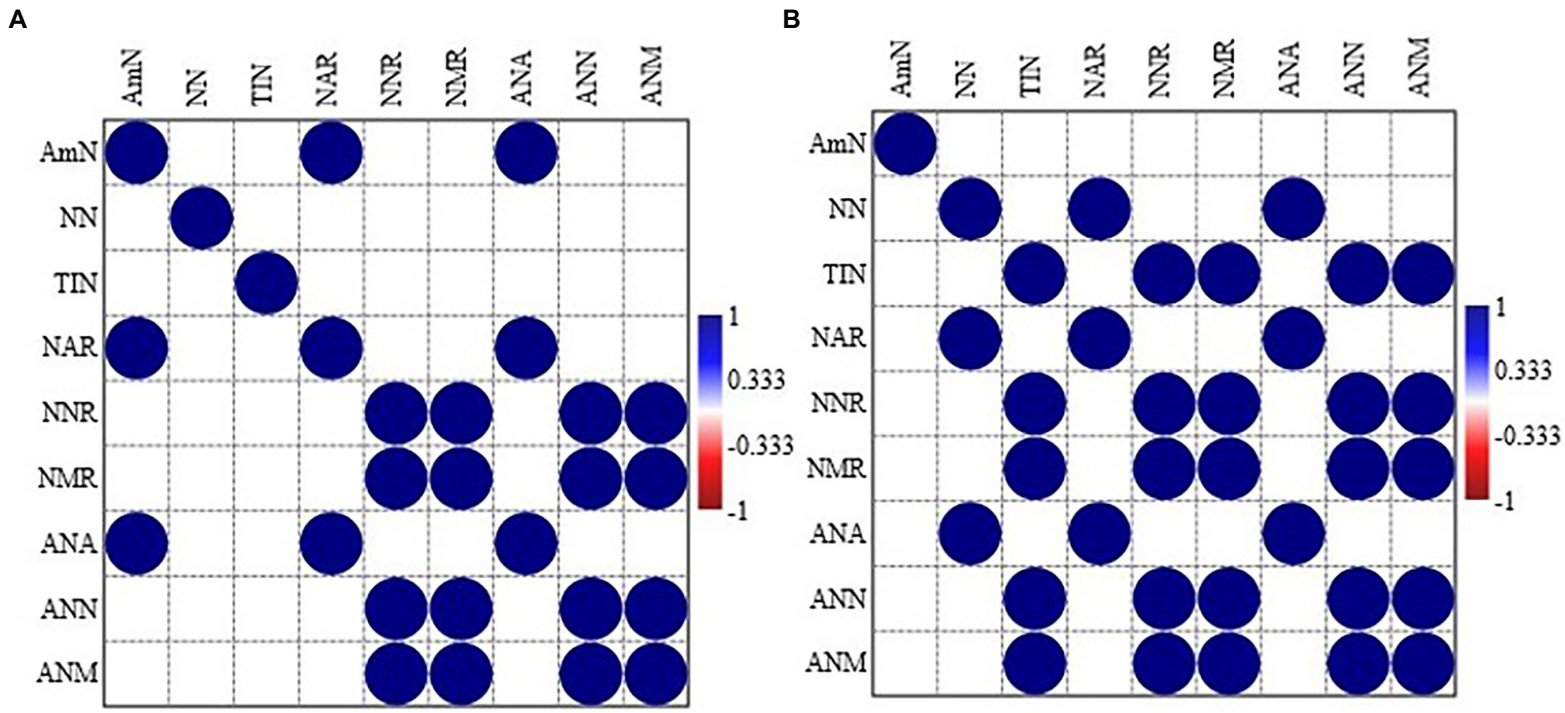
Pearson correlation between the different nitrogen forms in **(A)** Masson pine pure forest (MPPF) and **(B)** Masson pine and camphor tree mixed forest (MCMF) stand across all litter treatments. Pearson correlation is significant at *p* < 0.05; all the non-significant values are shown with blank box. AmN: NH_4_^+^-N, NN: NO_3_^−^-N, TIN, total inorganic nitrogen; NAR, net ammonium rate; NNR, net nitrification rate; NMR, net mineralization rate; ANA, annual NH_4_^+^-N production; ANN, annual NO_3_^−^-N production; ANM, annual total inorganic nitrogen.

**Figure 3:**
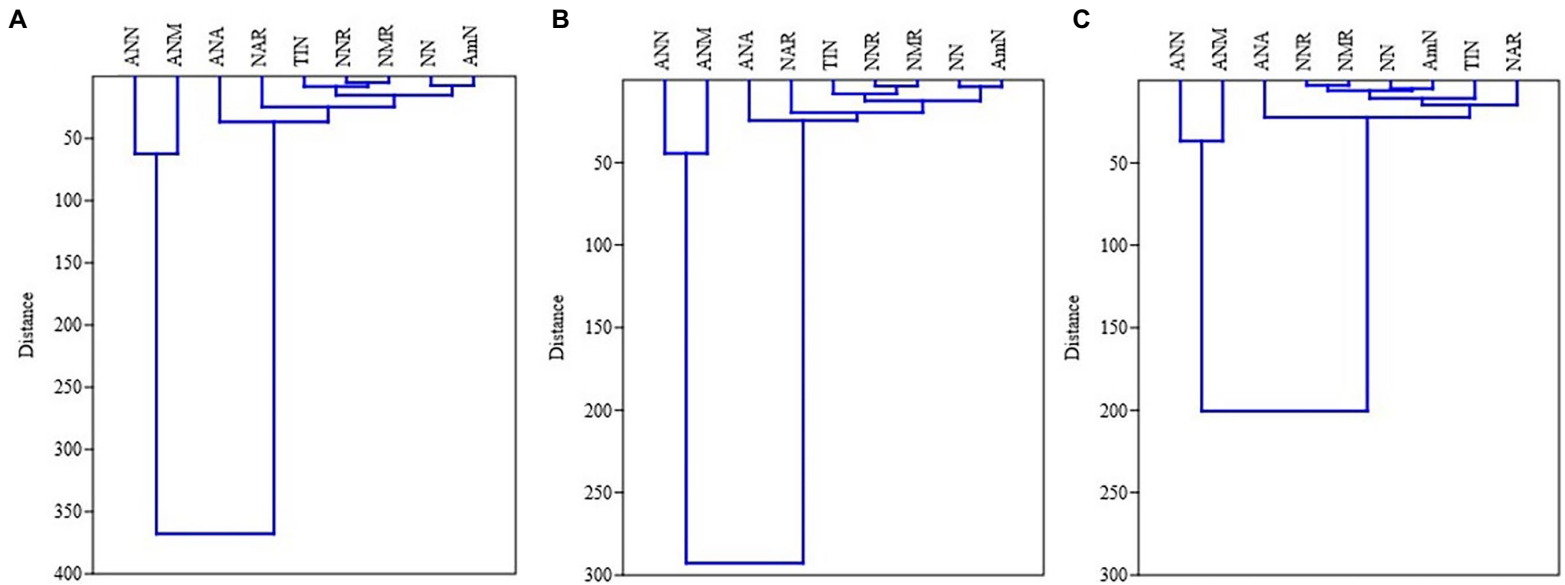
Correlation analysis – dendrograms showing hierarchical clustering of N variables in **(A)** LA: litter addition, **(B)** LC, litter control, and **(C)** LC: litter removal treatments across both forest types [MPPF and (B) MCMF stand]. AmN: NH_4_^+^-N, NN: NO_3_^−^-N, TIN, total inorganic nitrogen; NAR, net ammonium rate; NNR, net nitrification rate; NMR, net mineralization rate; ANA, annual NH_4_^+^-N production; ANN, annual NO_3_^−^-N production; ANM, annual total inorganic nitrogen.

In MPPF stand, NH_4_^+^-N was significantly correlated to net ammonium rate and annual ammonium N production. Net nitrification rate was significantly associated with net mineralization rate, annual NO_3_^−^-N, and annual total inorganic N production (Figure 2). In Masson pine and camphor tree mixed forest (MCMF) stand, NO_3_^−^N was significantly correlated to net ammonium rate and annual NH_4_^+^-N production. Total inorganic N content was significantly correlated to net nitrification, mineralization rate, and annual NH_4_^+^-N and NO_3_^−^-N production (Figure 2). Moreover, the cluster grouping (correlation) of all the N variables under different litter treatments is shown in (Figure 3).

## Discussion

N transformation rates are considered a valuable index of plant N availability (Schimel and Weintraub, 2003; Colman and Schimel, 2013), and litterfall plays a key role in the N transformation process. Litterfall input is generally an imperative way of nutrient transfer to forest soils (Vitousek et al., 1995). The quantity of litterfall varies significantly over a range of spatial and temporal scales and is determined mainly by climate, seasonality, topography, soil parent materials, and species distribution (Sayer, 2006). Sayer et al. (2007) mentioned that long-term litter addition increases soil bulk density, overland flow, erosion, and temperature fluctuations and upsets the soil water balance, causing lower water content during dry periods. Whereas, long-term litter removal severely depleted the forests of nutrients. The decline in soil N occurred over longer periods. Litter manipulation also increased or decreased large amounts of C, affecting microbial communities and altering soil respiration rates (Sayer et al., 2007).

In this study, litterfall manipulation treatments significantly altered soil N mineralization and nitrification in both subtropical forest types. N transformation differs with change in vegetation type because stand structure influences different environmental variables that directly impacts. Soil NH_4_^+^-N and NO_3_^−^-N contents were increased by approximately 57 and 35% in LA plots compared to LC plots in MCMF and about 19 and 14% in MPPF. LA treatment also increased soil annual net N mineralization in MPPF and MCMF compared to LC treatments in both forest types. Moreover, a strong positive correlation was observed between net N mineralization and nitrification rates (*p* = 0.001). The average proportion of nitrification rate to the net N mineralization rate was more than 80%. These results suggest that the N demand for soil microbes was sufficient (Schimel and Weintraub, 2003); thus, nitrification was dominant in the studied forest soils, especially in soils with a high-N mineralization rate (Son and Lee, 1997; Urakawa et al., 2016). A higher amount of soil N transformation in MCMF may contribute to the soil micro-environmental conditions, such as broadleaved camphor tree creating a shade condition to alter soil temperature and moisture than the MPPF. Soil moisture might also be influenced by canopy cover and stand density, which is often affected by tree species composition (Nadelhoffer et al., 2004; Aranda et al., 2012). Litter addition in forest soils also directly increases soil available nutrients, especially N and P (Schimel and Weintraub, 2003; Wang et al., 2013); therefore, another possible mechanism to explain this result was that added N can stimulate N processing when N availability is low but inhibit N processing when N availability is already high (Yoon et al., 2015; Gilliam et al., 2018).

LA treatment significantly increased soil NO_3_^−^-N content, while LR suppressed NH_4_^+^-N and NO_3_^−^-N content. The different responses of NH_4_^+^-N and NO_3_^−^-N concentrations in different forests might be attributed to the soil microbial communities’ composition, richness, species activities, and the C/N ratio of litter leaves in forests (Chen and Stark, 2000). Litter reduction was expected to reduce decomposition in mineral soil horizons due to the depression of microbial population and activities. LR treatment likewise decreased organic acid leaching from the litter layer. It reduced the substrate sources for microbial decomposition (Cullings et al., 2003) and changed soil temperature and soil moisture by increasing soil exposure (Fierer et al., 2005). In addition, the soil microflora and fauna complement each other in the comminution of litter, mineralization of essential plant nutrients, and conservation of these nutrients within the soil system (Marshall, 2000). LR treatment likely reduced microbial activity due to the removal of the substrate supply to the microbial community. Although complete removal of litter is unusual, substantial disruption of the litter layer is common in thinning operations, influencing water retention and flow, and reducing aeration and root penetration (Ballard, 2000).

A previous study found that soil N mineralization rate positively correlates with soil C/N ratio and SOM content (Yoon et al., 2015). Moreover, the litter addition increased soil mineralization, ammonification, and nitrification rates compared to the control plots because extra litter input in soils caused the decomposition of soil organic matter (SOM; Nadelhoffer et al., 1984). Evidence was very common and essentially a fertilizer effect on soil microbial communities in low-N and high-N soils (Morrison et al., 2016). These observations suggested that chronically elevated inputs of N over time can cause a convergence of rates and patterns of soil N processing on a landscape scale (Gilliam et al., 2018). N mineralization would be restricted to particularly N-rich microsites, and the mineralized NH_4_^+^-N would diffuse away from those microsites (Hart et al., 1994). If these N-rich sites last long enough, they might support the development of small nitrified populations, causing some limited nitrification to occur, though the overall soil condition would not appear conducive to nitrification.

The concentrations of NO_3_^−^-N and the annual gross NO_3_^−^-N accumulation in soil were much higher than NH_4_^+^-N content in two types of forest soils. This phenomenon should contribute to N absorption by plant roots in the soil. Plant roots generally uptake the NH_4_^+^-N easier than NO_3_^−^-N in forest soils. Moreover, an increase in the SOM and litter quantity enhances the soil activity, and this increment has direct involvement in the improvement of nutrient cycling and canopy development (Rothstein et al., 2004; Farooq et al., 2018, 2020; Rashid et al., 2020), along with greenhouse emission (Shakoor et al., 2020, 2021a,b), which, in return, has a positive impact on the ecosystem, plant growth, and overall SOC. In addition, tree species might directly affect N transformations through fine roots turnover in soil ecosystems (Hobbie, 2015; Urakawa et al., 2016; Wu et al., 2017).

## Concluding Remarks

Leaf litter quantity and quality influenced nutrient return to the soil. The current study found that NH_4_^+^-N and NO_3_^−^-N contents and annual net N mineralization in forest soils positively correlated with litter content proving litterfall as the major nutrient pathway and resource of forest soils. LA and LR treatments significantly affected N pools and N cycling in the mineral soil of the two types of forests. A large amount of soil N transformation in MCMF than in MPPF indicated that the optimal forest management in silviculture practices such as mixed forests should have higher nutrient return and accumulation into forest soils from the litterfall decomposition. The effects of different forest types with varying compositions of litter and chemical components on net N annual production in forest soils would provide a further understanding of N availability in forests. However, caution should be taken while applying our results, as many external factors that might influence the studied parameters in the open field conditions. Urban, suburban, and rural gradients can also be considered as these forests at different sites are influenced by the different intensities of anthropogenic activities, also include both lab-based and field-based experiments to complement the current results.

## Data Availability Statement

All the data has been used in the article. The raw data supporting the conclusions of this article will be made available by the corresponding authors on demand.

## Author Contributions

WY and XC: conceptualization and supervision. YC, YP, and XC: methodology. YC and WW: fieldworks and chemical analysis. YC, RS, and MUR: data analysis. WY, YC: writing original draft preparation. THF, SSA, and UK: writing – review and editing. All authors have read and agreed to the published version of the manuscript.

## Funding

This research was supported by the Key Research and Development Program of Hunan Province (2020NK2022), Joint Funds of the National Natural Science Foundation of China (grant number: U21A20187), the Scientific Research Fund of Hunan Provincial Education Department (18A150), and National Key R&D Program (grant number: 2020YFA0608100). The authors also thank Taif University Researchers Supporting Project number (TURSP-2020/38), Taif University, Saudi Arabia for supporting.

## Conflict of Interest

The authors declare that the research was conducted in the absence of any commercial or financial relationships that could be construed as a potential conflict of interest.

## Publisher’s Note

All claims expressed in this article are solely those of the authors and do not necessarily represent those of their affiliated organizations, or those of the publisher, the editors and the reviewers. Any product that may be evaluated in this article, or claim that may be made by its manufacturer, is not guaranteed or endorsed by the publisher.
